# Vitamins formed by microorganisms in fermented foods: effects on human vitamin status—a systematic narrative review

**DOI:** 10.3389/fnut.2025.1653666

**Published:** 2025-10-07

**Authors:** Erhan Keyvan, Elizabeth Adesemoye, Marie-Christine Champomier-Vergès, Emilie Chanséaume-Bussiere, Julie Mardon, Daniela Nikolovska Nedelkoska, Recep Palamutoglu, Pasquale Russo, Inga Sarand, Laurencia Songre-Ouattara, Biljana Trajkovska, Sibel Karakaya, Michail Syrpas, Christophe Chassard, Smilja Pracer, Guy Vergères, Daniel Heine, Christèle Humblot

**Affiliations:** ^1^Department of Food Hygiene and Technology, Faculty of Veterinary Medicine, Burdur Mehmet Akif Ersoy University, Burdur, Türkiye; ^2^Department of Microbiology, Faculty of Life Science, Federal University Oye Ekiti, Ekiti State, Nigeria; ^3^Université Paris-Saclay, INRAE, AgroParisTech, Micalis Institute, Jouy-en-Josas, France; ^4^Nutrifizz, Clermont-Ferrand, France; ^5^Université Clermont Auvergne, INRAE, VetAgro Sup, UMR 0545 Fromage, Lempdes, France; ^6^Faculty of Technology and Technical Sciences Veles, University St. Kliment Ohridski, Bitola, North Macedonia; ^7^Department of Nutrition and Dietetics, Faculty of Health Sciences, Afyonkarahisar Health Sciences University, Afyon, Türkiye; ^8^Department of Food, Environmental and Nutritional Sciences (DeFENS), University of Milan, Milan, Italy; ^9^Department of Chemistry and Biotechnology, Tallinn University of Technology, Tallin, Estonia; ^10^Centre National de la Recherche Scientifique et Technologique, Ouagadougou, Burkina Faso; ^11^Faculty of Biotechnical Sciences, University St. Kliment Ohridski, Bitola, North Macedonia; ^12^Department of Food Engineering, Faculty of Engineering, Ege University, Izmir, Türkiye; ^13^Department of Food Science and Technology, Kaunas University of Technology, Kaunas, Lithuania; ^14^Université Clermont Auvergne, INRAE, VetAgro Sup, UMR 0545 Fromage, Aurillac, France; ^15^Institute for Biological Research" Siniša Stanković", National Institute of the Republic of Serbia, University of Belgrade, Belgrade, Serbia; ^16^Agroscope, Bern, Switzerland; ^17^School of Agricultural, Forest, and Food Sciences, Bern University of Applied Sciences, Bern, Switzerland; ^18^Avignon Université, CIRAD, Institut Agro, IRD, Université de La Réunion, Montpellier, France; ^19^French National Research Institute for Sustainable Development (IRD), Montpellier, France

**Keywords:** B vitamins, fermented foods, human intervention studies, microbial vitamin formation, vitamin bioavailability, vitamin K2

## Abstract

**Introduction:**

Vitamin deficiencies remain a global health issue, particularly among vulnerable populations. As microorganisms also produce vitamins, this has led to considering Fermented Foods (FF) as potential vehicles for improving vitamin intake. This systematic narrative review, which exclusively relies on human studies, aims to assess the extent to which the consumption of vitamin-rich FF contributes to the maintenance or enhancement of vitamin status in healthy or deficient populations.

**Methods:**

A comprehensive literature search (1970–2024) was conducted following the protocols of EFSA and the COST Action PIMENTO to identify interventional and observational studies investigating the influence of FF on biomarkers of vitamin status.

**Results:**

Findings confirm that certain microorganisms, including *Bacillus subtilis, Propionibacterium freudenreichii*, and some lactic acid bacteria, can increase the levels of vitamins K2, B2, B9, and B12 in FF. Evidence of bioavailability and physiological effects is reported. Notably, folate (vitamin B9) bioavailability was enhanced in some cases following the consumption of Camembert cheese naturally rich in folate, while vitamin K2 status was effectively improved in several studies on natto (fermented soy) and in one study on Jarlsberg cheese. However, evidence for other B vitamins (B1, B2, B3, B5, B6 and B12) is limited or inconsistent, and no human evidence exists for other vitamins. Vitamin bioavailability was found to be significantly influenced by the food matrix, fermentation type, microbial strain, and the form of the vitamin (vitamers). Effects may also be influenced by interactions with gut microbiota, including microbial vitamin synthesis and modulation of absorption.

**Discussion:**

Despite encouraging data, there is a lack of well-controlled, large-scale human studies to validate FF as a sustainable strategy to improve vitamin status. Future human studies research should investigate strain-specific effects, food matrix interactions, and long-term health outcomes.

## 1 Introduction

Vitamin deficiencies have severe health consequences worldwide (1). Vitamins can act as cofactors, signaling molecules, or gene regulators, thus affecting metabolic pathways, enzymatic reactions, or cellular homeostasis. They are categorized into water-soluble and fat-soluble vitamins, and each has a specific role, which is presented in this review.

Vitamin requirements are higher for young children, pregnant and lactating women as well as women of reproductive age, adolescents, and older adults, who are therefore particularly at risk of deficiencies (2). The prevalence of individual vitamin deficiencies is poorly quantified, due to undiagnosed cases resulting from unclear symptoms and the non-inclusion of biomarkers for measuring vitamin status in population studies (1). Micronutrient malnutrition affects approximately from 2 billion up to 4–5 billion people worldwide (3, 4). Aside from specific populations with malabsorption related to disease or treatment, the primary cause of vitamin deficiencies is inadequate intake (5).

Common strategies to combat deficiencies include dietary diversification, fortification of staple foods, and supplementation (1). Although the effectiveness of these strategies is well established, the global prevalence of micronutrient deficiencies is still high and varies depending on contextual factors (1, 4). Dietary diversification involves consuming a variety of nutritionally rich foods, but this may be limited for some populations due to cost or dietary choices, such as excluding animal-based foods, which are the primary source of vitamin B12 (6). Fortification of staple foods and supplementation using capsules, tablets or drops relies on synthetic forms of vitamins, which may be unaffordable or inaccessible to vulnerable populations in resource-limited regions (7). In addition, the consumption of synthetic forms of vitamin B9 can mask symptoms of vitamin B12 deficiency and lead to irreversible neuropathy (2). Therefore, exploring additional ways to improve vitamin intake to complement existing strategies is essential.

Fermented foods (FF) are a particular category of foods, which are produced from various materials, through microbial action. FF are estimated to account for around 30% of the foods consumed worldwide (8). FF are highly diverse and are prepared from all types of substrates (milk, cereals, fruits, etc.), using different processes, different microorganisms (spontaneous fermentation, back-slopping, or inoculation with defined starter strains), and different fermentation types (lactic, acetic, etc.) resulting in a wide variety of food products consumed as staples, beverages or condiments (9). They may therefore significantly contribute to consumers’ vitamin intake.

Among microorganisms responsible for fermentation, some have been demonstrated to possess vitamin-forming capabilities and have been successfully utilized to enhance the vitamin content of raw materials (10). However, the vitamin production capacity drastically differs, and not all vitamins are present in FF; with their formation depending on the microbial strain and fermentation conditions. Among the fat-soluble vitamins, microbial synthesis of vitamin K2 (menaquinones) in FF is the most studied. Vitamin K2, which differs structurally and functionally from vitamin K1 (a plant-derived phylloquinone), plays a key role in blood coagulation and bone metabolism by activating vitamin K-dependent proteins. Various FF such as fermented soybeans (natto, cheonggukjang) and cheeses are valuable dietary sources of vitamin K2, with microorganisms such as *Bacillus subtilis* responsible for its formation (11).

Among water-soluble vitamins, the ability of lactic acid bacteria (LAB) to synthesize different B-group vitamins have been studied, with particular emphasis on folate (vitamin B9) and riboflavin (vitamin B2) and to a lesser extent on cobalamin (vitamin B12), which is primarily associated with *Propionibacterium freudenreichii* (12, 13). B-group vitamins are essential for energy metabolism, DNA synthesis, neurotransmitter function, and the regulation of homocysteine. They act as enzyme cofactors in metabolic pathways. They are found in various FF, including dairy products (14).

Other vitamins, such as the fat-soluble vitamins A, D and E, as well as the water soluble vitamin C (ascorbic acid), are rarely studied in the context of FF. Vitamin A is involved in vision, immune function, and cellular differentiation through gene regulation (15). Vitamin D, regulates calcium and phosphate metabolism, essential for bone health and immune function through nuclear receptor activation (16). Vitamin E functions as an antioxidant, protecting cell membranes from oxidative damage and influencing lipid metabolism (17). Similarly, vitamin C (ascorbic acid), which also functions as an antioxidant, enhances iron absorption, supports collagen synthesis, and plays a role in immune regulation (18).

Some microorganisms are capable of producing precursors to vitamins A, D, and E, or industrially relevant forms of vitamin C; however, these pathways are uncommon or absent in bacteria present in traditional FF (19, 20). Vitamin C and E are major representatives of industrially produced vitamins (21). The production of vitamin E is chemical and/or using engineered microorganisms and little is known about the localization of the tocopherol biosynthetic enzymes in bacteria. Photosynthetic bacteria such as *Synechocystis* spp. have contributed to the identification of several tocopherol pathway genes (22). Vitamin C can be produced by some bacteria, but not usually those involved in food fermentation, and often by genetically engineered strains. Therefore, its formation is generally not applicable in FF (23). In fermented vegetables such as sauerkraut, vitamin C is present in meaningful amounts, but it originates primarily from the raw cabbage and not from microbial synthesis (114). In some commercial products, acerola powder or other fruit extracts may be added to boost vitamin C content, but this should not be attributed to the fermentation process itself.

The incorporation of vitamin-producing microorganisms in food fermentation can enhance the nutritional quality and offers a sustainable, environmentally friendly alternative to chemical synthesis. Ongoing research on novel microbial sources and optimization strategies indicates that microbial vitamin production could play a crucial role in enhancing global nutrition security (24). Many articles explore the use of microorganisms to increase vitaminic content of various raw materials and food, with several reviews comprehensively summarizing their findings (11, 12, 25). However, the absorption and physiological effects of naturally occurring forms of vitamins (vitamers) differ depending on factors such as food matrix and fermentation conditions. Whether FF can help maintain adequate vitamin status in healthy individuals, or improve status in deficient populations, remains insufficiently studied.

Regarding hosts, microorganisms from the gut microbiota may also contribute to the host’s vitamin status through direct vitamin synthesis in the digestive tract or by improving vitamin bioavailability (26). FF can modulate gut microbiota composition and function, potentially enhancing its contribution to vitamin metabolism. In addition, FF can serve as a source of microorganisms with vitamin-synthesizing capabilities, which may continue to produce vitamins directly in the gut during gastrointestinal transit (26). The contribution of gut microbes to vitamin status is insufficiently studied and the effect of FF consumption on the contribution of the gut microbes is inexistent. Nevertheless, some hypothesis have been formulated by several authors, and will be presented in the manuscript.

The objective of this systematic narrative review was to assess whether the consumption of FF contributes to the vitamin status of both healthy and vitamin-deficient populations. This review is part of a series of articles of the COST Action CA20128—Promoting Innovation of ferMENTed fOods (PIMENTO) on the health benefits and risks of FF (27). A unique aspect of this work is that it includes only human studies, evaluating the effects of FF in which vitamins were formed through fermentation, based on changes in vitamin levels or related biomarkers in the body. In addition, all types of FF and all types of vitamins were considered, ranging from well-studied dairy products to fermented soybeans and cereal grains. The potential link between FF consumption, gut microbiota modulation, and vitamin status is briefly discussed, though not systematically addressed in the included studies due to scarcity of related literature.

## 2 Methods

### 2.1 Study design and protocol

This systematic narrative review was conducted in accordance with the indicative guidance of the European Food Safety Authority (EFSA) for evaluating mechanisms of action and follows a predefined protocol aligned with the PROSPERO format, the PIMENTO Study Protocol, and the 24-step methodological framework proposed by Muka et al. (27, 28). The protocol was registered in the Open Science Framework (OSF) using the Open-Ended Registration modus (29) and is available at https://osf.io/8psdz/. The timeline for the review extends from September 1, 2023, to December 31, 2024.

### 2.2 Literature search

**Figure 1:**
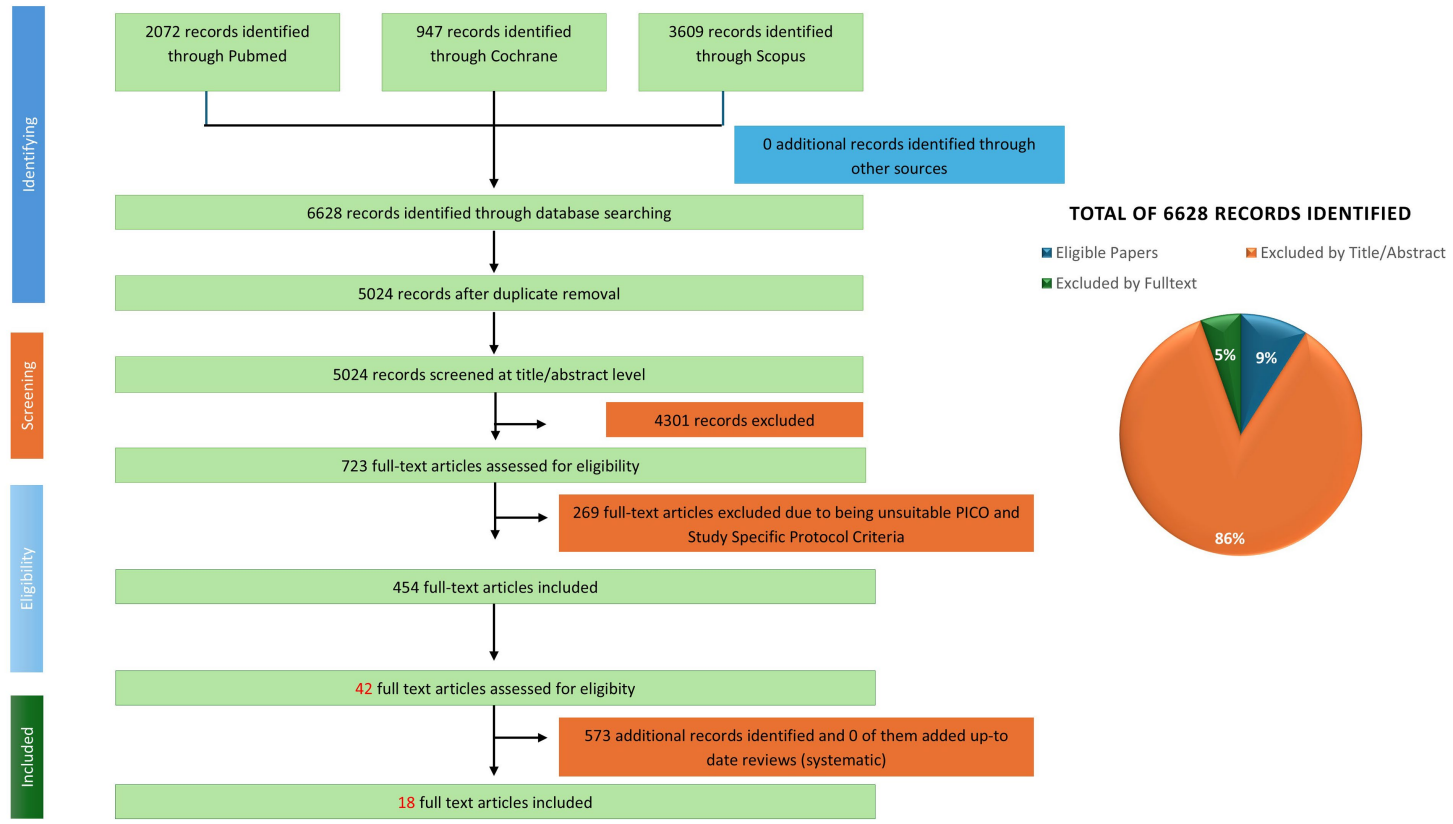
PRISMA flow diagram illustrating the study selection process and results of the systematic literature search.

A systematic search was conducted across PubMed, Scopus, and the Cochrane Library covering publications. In two phases: first from January 1, 1970, to August 31, 2023, in English, then, an extra search that extend August 2023 to December 2024. The search utilized a generic string developed by the University of Zurich Library for PIMENTO, covering the maximum diversity of FF groups, human studies, and dietary intake terms. Functional terms related to vitamin metabolism were added to the generic PIMENTO search string. In addition to the PIMENTO search strings, search terms specific to the review were created to encompass the functional aspects of the study, including clinical indications and biological activities associated with vitamin status. Studies involving both healthy and vitamin-deficient populations (including children) were included, as vitamin deficiency is particularly relevant in younger populations. The systematic review encompasses human intervention studies, including randomized controlled trials, non-randomized controlled studies, and various other intervention studies, as well as observational studies such as cohort studies, case–control studies, and cross-sectional studies, in accordance with the EFSA guidance (30). Systematic reviews, whether including meta-analysis or not, were screened to identify potentially relevant studies. Animal and *in vitro* studies were excluded from the systematic review process. The selection of studies and extraction of data adhered to the procedures specified by Muka et al. and the Cochrane Handbook (28, 31). The CADIMA software assisted the organization and screening of references, thereby ensuring a systematic and reproducible selection process (32). The search and study selection strategy were documented with a PRISMA flow diagram to illustrate the inclusion and exclusion process (Figure 1).

### 2.3 Study selection criteria

The selection of studies was based on predefined inclusion and exclusion criteria, aligned with the PIMENTO Study Protocol. The study population, intervention/exposure, and comparator conditions were established using the PI(E)COS framework to ensure the relevance and quality of the studies included.

#### 2.3.1 Inclusion and exclusion criteria

##### 2.3.1.1 Population

The review encompassed individuals of all ages, both healthy and those with vitamin deficiencies. Studies involving non-healthy populations, with the exception of those specifically exhibiting vitamin deficiencies, were excluded. Individuals aged 18 and older were classified as adults; however, children were also included due to their increased susceptibility to vitamin deficiencies.

##### 2.3.1.2 Intervention or exposure

This review analyses the intervention/exposure of FF consumption, as stated in the PIMENTO search string, encompassing the following food groups: dairy products, meat and fish, fruits and vegetables, beverages, legumes, cereals, and grains.

Exclusion criteria: FF fortified with synthetic forms of vitamins (e.g., fermented milk fortified with vitamin D). Alcoholic beverages containing more than 1.25% alcohol by volume. Research on probiotics as supplements suggests that their effectiveness is contingent upon their addition at the onset of the fermentation process, where they contribute to the fermentation process. Studies contain prebiotic fibers or additional bioactive compounds as potential confounding variables. Applications of FF beyond nutrition, such as topical or nasal uses. Interventions could be standalone or combined with other dietary components, provided that non-fermented control conditions were appropriately managed.

##### 2.3.1.3 Comparator or control

The control groups included: Non-consumption or lower consumption of the FF in question. Consumption of the non-fermented version of the food(s). Any suitable non-fermented placebo or control.

A two-step approach for study selection was employed: Studies were first evaluated according to the PI(E)O criteria, which encompass Population, Intervention/Exposure, and Outcome. The comparator conditions were subsequently evaluated independently. This method facilitated a thorough assessment of the control groups in the chosen studies, while also highlighting deficiencies in the quality of control measures across the studies.

##### 2.3.1.4 Outcomes

The primary outcome of this review is an increase in vitamin levels in the studied population after consuming FF. The evaluation of vitamin status was be conducted using specific biomarkers for each vitamin. For example, serum folate concentration was used as a biomarker for folate status.

Furthermore, safety outcomes were evaluated, encompassing any adverse effects or risk indicators linked to the intake of FF, as detailed in Appendix B of the EFSA guidance (30).

P = All healthy or vitamin deficient population, all ages
I = Any FF
C = Non-consumption or lower consumption of the FF in question. Consumption of the non-fermented version of the food(s). Any suitable non-fermented placebo or control
O = Increase in vitamin levels.

### 2.4 Study selection and data extraction

The data extraction process followed the methodology outlined in the PIMENTO Study Protocol and was conducted in accordance with the systematic review guidelines by Muka et al. (28). The following steps were implemented to ensure accuracy and consistency:

#### 2.4.1 Study selection

Initial screening of titles and abstracts by at least two independent reviewers. Retrieval of full-text articles and application of inclusion/exclusion criteria. Resolution of discrepancies through discussion or consultation with a third reviewer.

#### 2.4.2 Data extraction and organization

The specific data extraction form, a modified version of the PIMENTO WG3 data extraction form was developed using guidance from the Cochrane Handbook for Systematic Reviews of Interventions (31), the EFSA guidance on health claims (30) (Appendix B), and the STROBE statement for observational studies (33). These forms were used to collect structured data from each eligible study, including study design, population demographics, health and vitamin status, intervention characteristics (type, dosage, frequency), comparator details, primary and secondary outcomes (e.g., vitamin biomarkers), and indicators of bias and study quality.

#### 2.4.3 Systematic selection and screening software

Screening and reference management were conducted using the CADIMA software, where a consistency test was applied to a subset of the dataset (32). In cases of inconsistency, the study protocol and selection strategy were refined, including the use of a third reviewer when necessary, to improve reproducibility and systematic accuracy. The PRISMA flow diagram was used to document the literature selection process transparently (Figure 1).

### 2.5 Non-systematic review components

In line with the PIMENTO S2 Study Protocol, this review includes a non-systematic component to complement the systematic findings. It summarizes the characteristics of the FF, including their microbial strains and food matrices. Additional supportive evidence from the literature addresses the bioavailability of vitamins in FF, their mechanisms of action such as microbial biosynthesis and gut microbiota modulation, and the safety aspects, including microbial safety and potential adverse effects.

## 3 Results and discussion

This review analyzed 18 studies on health responses in humans following the consumption of FF (Figure 1). Table 1 provides an overview of the vitamins present in FF, their effects, biomarkers and role of food and gut microbiota. Acting as cofactors, signaling molecules, or gene regulators, vitamins influence metabolic pathways, enzymatic reactions, and cellular homeostasis.

**Table 1:** Overview of water- and fat-soluble vitamins relevant in FF, (A, D, K, B1, B2, B3, B5, B6, B9, B12), including their absorption, metabolic activation, physiological roles, biomarkers, gut microbiota interactions, and microbial contributions.

Vitamin	Absorption	Metabolic Activation	Physiological Roles	Markers	Interactions with Gut Microbiota	Food Microbial Contribution
Vitamin A (Retinol, β-carotene)	Requires bile salts for uptake; influenced by food matrix (e.g., cooked foods improve bioavailability)	Pro-vitamin A (β-carotene) converted to retinal and retinoic acid in the intestine and liver	Vision (via rhodopsin) Immune modulation Cellular differentiation and reproduction	Serum retinol and retinyl esters	Microbiota modulates bile acid metabolism, aiding fat-soluble vitamin absorption; microbiota composition may influence immune signaling and vitamin A metabolism	Fermentation may stabilize carotenoids in food matrices, potentially improving bioavailability
Vitamin D (Cholecalciferol)	Absorbed with dietary fat; synthesized in the skin under UVB exposure	Hydroxylated in the liver to 25-hydroxyvitamin D (25-D) and in the kidney to 1,25-dihydroxyvitamin D (1,25-D) by cytochrome P450 enzymes	Calcium and phosphorus homeostasis Immune modulation (via the vitamin D receptor, VDR) Cell proliferation and apoptosis regulation	Serum 25-hydroxyvitamin D	Microbial metabolites like short-chain fatty acids (e.g., butyrate) upregulate VDR expression; some gut bacteria compete with vitamin D for binding to VDR	Fermentation influences the stability of vitamin D in fortified foods but *does not produce* it directly
Vitamin K (K1: Phylloquinone, K2: Menaquinones)	Fat-soluble; requires bile salts and dietary fat for absorption; stored in the liver	Reduced to hydroquinone by vitamin K epoxide reductase (VKOR) for activation of γ-carboxylase enzymes	Activates clotting factors (II, VII, IX, X) Modifies osteocalcin for bone health Prevents arterial calcification by activating matrix Gla-protein (MGP)	Prothrombin time, serum uncarboxylated osteocalcin (ucOC), serum MGP, plasma phylloquinone and menaquinone levels	Gut bacteria (e.g., *Bacteroides*) produce menaquinones (vitamin K2 forms); gut microbiota modulation influences systemic vitamin K status	Production of menaquinones MK-7 (from *B. subtilis* in natto); MK-9 (from *P. freudenreichii* and selected LAB in aged cheese); both highly bioavailable; fermentation *does not produce* vitamin K1 (phylloquinone), but may enhance its bioaccessibility in plant-based foods.
Vitamin B1 (Thiamine)	Water-soluble; hydrolyzed by intestinal phosphatase and absorbed in the small intestine	Converted to thiamine pyrophosphate (TPP) by the enzyme thiamine diphosphokinase in the bloodstream	Glycolysis Krebs cycle Pentose phosphate pathway. Tricarboxylic acid cycles	Thiamine pyrophosphate in whole blood	*Bacteroides* and *Fusobacteria* are the most common phyla able to synthesize TPP in the gut. However, some thiamine-auxotrophic species, such as *Ruminococcaceae*, can compete for this vitamin, especially in the small intestine. *Bifidobacterium* spp. are associated with the biosynthesis of folate, niacin, pyridoxine, and thiamine	LAB fermentation increases the level of thiamine in cereal-based products, cassava root, soy milk, vegetable such as amaranth leaves and locust bean.
Vitamin B2 (Riboflavin)	Water-soluble; hydrolyzed by intestinal phosphatases before absorption in the small intestine via carrier-mediated saturable transport at the brush-border membrane of villus cells	Flavokinase phosphorylates riboflavin into flavin mononucleotide (FMN), which is then further converted to flavin adenine dinucleotide (FAD) by FAD synthetase.	Energy production Cellular function Growth, and development Metabolism of fats, and steroids Metabolism of vitamins B3 and B6 Maintain normal levels of homocysteine, an amino acid in the blood	Erythrocyte Glutathione Reductase Activity Coefficient (EGRAC). EGRAC measures the activity of glutathione reductase in red blood cells before and after exposure to riboflavin (as FAD).	*Bacteroides* species, *P. copri*, and *E. rectale*, produce wide-ranging B-vitamin, while *F. prausnitzii* and *Ruminococcus gnavus*, significantly contribute to the biosynthesis of cobalamin (B12), niacin (B3), and riboflavin (B2)	Fermentation significantly enhances dietary riboflavin in foods like sourdough, cereal-based formulation, plant-based beverages
Vitamin B3 (Niacin)	Water-soluble; both the acid and amide forms are absorbed via a combination of sodium-dependent facilitated diffusion and passive diffusion, depending on the concentration, in the small intestine, with some absorption occurring in the stomach.	All tissues in the body convert absorbed niacin into the coenzyme nicotinamide adenine dinucleotide (NAD), that can be further converted into nicotinamide adenine dinucleotide phosphate (NADP), in all tissues except skeletal muscle	Catabolic reactions that transfer the energy to adenosine triphosphate (ATP) Maintenance of genome integrity Control of gene expression, and cellular communication Maintaining cellular antioxidant function	Assessing urinary metabolites, 1-methylnicotinamide (1-MN), and 1-methyl-2-pyridone-5-carboxamide (2-PYR).	Bifidobacterium spp. are associated with the biosynthesis of folate, niacin, pyridoxine, and thiamine	Fermentation increases the level of vitamin B3 in products based on rice, maize, sorghum, millet, cassava
Vitamin B5 (Pantothenic Acid)	Water-soluble; hydrolyzed by intestinal pyrophosphatases and phosphatases before absorption in the small intestine via passive and sodium-dependent facilitated diffusion (concentration-dependent),	Converted into CoASH or peptide-bound 4′-phosphopantetheine	Energy generation Cell growth Neurotransmitter synthesis Synthesizing hormones Maintaining optimal conditions for skin, hair, and nails.	Enzymatic hydrolysis of bound pantothenate in whole blood and erythrocytes	*Bacteroides*, *Prevotella*, *Klebsiella* spp. contribute to microbial pantothenate biosynthesis	Fermentation can enhance the vitamin B5 content of maize-, sorghum-, rice-based products
Vitamin B6 (Pyridoxine)	Water-soluble; Pyridoxine, pyridoxamine, and pyridoxal are absorbed by mucosal cells of the small intestine via passive diffusion, while their phosphorylated analogs first undergo dephosphorylation; stored primarily in the liver, with smaller amounts in the brain and muscles	Vitamin B6 has three natural forms: pyridoxine (PN), pyridoxal (PL), and pyridoxamine (PM), all of which converted into the coenzyme pyridoxal 5-phosphate (PLP or P5P).	Amino acid, protein, carbohydrate, and lipid metabolism Neurotransmitter synthesis Glycogenolysis Gluconeogenesis	Erythrocyte transaminase activities; plasma levels of metabolites involved in PLP-dependent reactions, such as the kynurenine pathway, (cystathionine), and glycine decarboxylation	Bifidobacterium spp. are associated with the biosynthesis of folate, niacin, pyridoxine, and thiamine	Yeast and LAB fermentation enhance the vitamin B6 content in foods like cereal-base products and vegetable juice.
Vitamin B9 (Folate)	Water-soluble; absorbed as monoglutamate in the small intestine	Absorbed as monoglutamate; converted to dihydrofolate and thento tetrahydrofolate (THF) for one-carbon metabolism	DNA and RNA synthesis Homocysteine metabolism Essential for cell division	Plasma total folate, red blood cell (RBC) folate levels	Gut bacteria like those from the genera *Lactobacillus sensu lato* and *Bifidobacterium* produce folate, contributing to systemic levels	Fermentation significantly increases folate content in foods such as yogurt, sauerkraut, and sourdough
Vitamin B12 (Cobalamin)	Water-soluble requires intrinsic factor for absorption in the ileum	Converted to methylcobalamin (for homocysteine metabolism) and adenosylcobalamin (for energy metabolism)	DNA synthesis Neurological health Energy production	Serum B12 levels, methylmalonic acid (MMA), homocysteine	Produced in the colon by certain gut bacteria, but absorption is limited to the ileum; overall B12 status depends on gut health and intrinsic factor availability	Produced by bacteria (*Propionibacterium freudenreichii*) in specific FF like Swiss cheese and fortified plant-based alternatives

From references (2, 6, 15, 16, 34, 35, 39, 57, 64, 65, 67, 72–77, 81).

The detailed presentation of the respective observational or interventional studies is presented in the relevant sections of this review, organized by individual vitamin (Table 2). To provide context for the observed effects, the following section introduces the food source of the vitamin, the underlying mechanisms of action of the vitamins being investigated and a presentation of human studies.

**Table 2:** Effect of the consumption of FF fortified in vitamin K and B through fermentation on vitamin status and related health outcomes in human.

FF	Vitamin	Microorganism responsible for vitamin synthesis	Study design	Duration of the study	Country	Population/age/sex	Sample size	Health status	Comparison/Control	Dose, delivery and timing	Outcome/biomarker	Effect	References
Vitamin K													
Natto	K1/K2 (MK-4, MK-7)	NS	Interventional -Clinical trial	72-h monitoring after meal consumption	Netherland	Adults/~34 years/Male	6	Healthy	Control: pure vitamin K1 or spinach (vitamin K2)	Vitamin K1 3.5 μmol 400 g Spinach 3.5 μmol vitamin K1 200 g Natto 3.1 μmol vitamin K2	Serum Vitamin K1 and K2 concentration	Natto ↑Vitamin K2 10 times higher than vitamin K1 concentration after eating spinach	(49)
Natto	K2 (MK-7)	*B. subtilis natto*	Observational	unknown	Japan	Adults/25–34 years/Male and Female	134	Healthy	Different natto intakes frequency	rare occasional (a few times a month) -requent (a few times a week)	Serum concentrations: -MK-7 -Gla-OC -Glu-OC	Male and female: higher MK-7 and lower Glu-OC Male: higher Gla-OC	(50)
Natto	K2 (MK-7)	*B. subtilis natto*	Interventional	3 × 7 days	Japan	Adults/25–34 years/Male and Female	8	Healthy	Control: baseline and regular natto consumption	[vitamin K2]: Regular natto: 775 μg/100 g Reinforced natto: 1,298 μg/100 g 1,765 μg/100 g	Serum concentrations: -MK-7 -Gla-OC -Glu-OC	↑ MK-7 and Gla-OC ↓ Glu-OC	(50)
Natto	K1/K2 (MK-4, MK-7)	*B. subtilis* OUV 3481	Interventional	14 days	Japan	Adults/ 24–46 years/ male and female	48	Healthy	Regular vs. reinforced natto with ↑ MK-7	3 groups Regular natto (865 μg MK-7/100 g) Reinforced natto (1,295 or 1,730 μg MK-7/100 g) 50 g/day for 14 days	Serum concentrations: -MK-7 -Gla-OC	All natto: ↑ serum MK-7 Reinforced natto: ↑ Gla-OC Regular natto: no sig. Effect on Gla-OC	(51)
Natto	K1, K2	NS	Observational cross-sectional	2007 and 2008	Japan	Elderly/>65 years /male	1,662	Healthy	Four natto intake groups: <1, 1, several, or ≥1 pack/day (40 g/pack).	Self-reported natto intake (40 g = 20 μg K1 + 380 μg K₂) via FFQ.	-Bone mineral density -serum Glu-OC	High natto intake: -↑ Bone mineral density ↓ Glu-OC	(39)
Natto	K1/K2 (MK-4, MK-7)	NS	Observational	7 days	Japan	Adults/~23 years/male and female	41	Healthy	High and low natto consumers	7d food record fasting	Blood coagulation markers	Natto improves blood coagulation markers	(48)
Natto	K2	NS	Interventional	1 year	Japan	Adults/~34/female	73	Healthy/ premenopausal	Four groups consuming or not natto at different frequency	Group 1: never Group 2: once/month Group 3: once/week Group 4: 3×/week at lunch for 1 year	BAP, Glu-OC, stiffness index	Group 4: ↑ BAP, ↓ Glu-OC at 6 mo vs. other groups; no Δ in stiffness index; reduced risk of ↓ bone formation markers (OR = 0.07)	(47)
Natto	K1/K2 (MK-4, MK-7)	*B. subtilis*	Interventional	3 days	Japan	Adults/21–60 years/male and female	32	Patients taking warfarin for thrombose prevention	Natto, vitamin K syrup	50 g/d, 3 days consumption of boiled natto (to decrease the content of living *Bacillus subtilis*)	Plasma levels of Vitamin K (phylloquinone, MK-4, MK-7)	↓ plasma vitamin K after boiled natto intake compared to natto and vitamin K syrup	(46)
Jarlsberg Cheese	K1/K2(MK-4, MK-7, MK-8, MK-9, MK-9 (4H))	*P. freudenreichii*	RCT – crossover (3:2)	12 weeks (2 × 6 weeks)	Norway	Adults/~33 years/female	66	Healthy	Camembert group (no K2) switched to Jarlsberg at week 6	57 g/day Jarlsberg, 25–50 g/day Camembert, daily	PINP, tOC, cOC, Glu-OC, RO, MK-9(4H), CTX, lipids, HbA1c, Ca++, Mg++	Jarlsberg ↑ PINP, tOC, cOC, RO, MK-9(4H); ↓ HbA1c, Ca++, Mg++; Camembert = no effect; switching to Jarlsberg: similar improvements	(53)
Jarlsberg Cheese	K1/K2(MK-4, MK-7, MK-8, MK-9, MK-9 (4H))	NS	Interventional (Response Surface Pathway design)	12 weeks (3× 4 weeks)	Norway	Adults/18–25 years/ male and female	20	Healthy, endurance-trained	Within-subject dose escalation	3 escalating dose levels (starting: 47 g/day; estimated OED: 73 g/day F, 84 g/day M); Jarlsberg cheese replacing other cheeses	MK-7 to MK-9(4H), cOC, Glu-OC, OC ratio, PINP, BAP, CTX, BMD (L1–L4), VO₂max, RMR, strength, HbA1c	↑ OC, ↑ BMD, ↓ BTMs initially → ↑ at final; ↑ VO₂max, ↑ RMR, ↑ strength; ↓ s-Ca, ↓ s-Mg	(52)
Vitamin B9													
Cheese	B9	NS	Interventional-Single-center RCT: Balanced 4-treatment, 4-period, crossover	24 h	Germany	Adults/~24 years/male and female	24	Healthy	PGA solution	Camembert (200 g, 1,294 folates) wheat germ (50 g, 534 nmol folates) spinach (500 g, 1,185 nmol folate) PGA (95 mM, 852 nmol folates)	Plasma and urine concentrations of 5-CH3-H4 folate	Bioavailability of 5-CH₃-H₄ folate: -PGA 21.8% -Spinach: 6.8% -Wheat germ 2.7% -Camembert: 1.7%	(60)
Cheese	B9	NS	Interventional randomized cross over.	24 h	Germany	Adults/~26 years/Female	2	Healthy,	PGA solution	Camembert (200 g, 448 nmol) PGA (453 nmol)	Folate bioavailability	-Bioavailability (64%)	(59)
Tea	B9	NS	Observational–cross sectional	24 h dietary recall	China	Adults/18–30 years/Female	2,630	Healthy	No tea drinkers	No tea drinkers Weekly tea drinkers Daily tea drinkers	Plasma folate concentration	Han population: ↑ 6.8% plasma folate concentration in weekly tea drinkers -↑7.1%in daily tea drinkers	(63)
Other vitamin B													
Miscellaneous	B3	NS	Observational-Prospective cohort study	<180 days	Netherlands	Adults/20 to 70 years/Male and Female	485–492	All population	Not applicable	FFQ and 24 h recalls	Plasma and urine metabolomes	Vitamin B3 as a biomarker of coffee intake	(82)
Dairies	B3, B5	NS	Observational–Cohort study	Not applicable	USA	Adults/40 to 55 years/male and female	3,712 and 4,078	Healthy	Quartiles of category of dairy intakes	FFQ	Plasma metabolomes	Higher dairy intake linked to vitamin B5 and B3	(83)
Probiotic yogurt	B1, B2, B6	*S. thermophilus* *L. acidophilus* *L casei* GG	Interventional	1 month	Austria	Adults/25–36 years/male and female	12	Healthy	Heat treated yogurt	2-week consumption of 500 g/day thermally inactivated yogurt, then 2-week consumption of 500 g/day yogurt	Plasma, Urine, Fecal vitamin B1, B2 and B6	Heat treated yogurt: -plasma and urine ↑vitamin B1 -plasma alteration of vitamin B2 metabolites (↑ FAD), ↓FMN -↑vitamin B6 Yogurt: -plasma vitamin B1, B2, B6 No effect on fecal excretion	(84)
Probiotic yoghurt	B1, B2, B6	*S. thermophilus* *L. acidophilus* *L casei* sp. casei	Interventional	1 month	Austria	Young/22–29 years/female	33	Healthy	Conventional yogurt	2 groups consuming conventional of probiotic yogurt: 2-week consumption of 100 g/d, then 2-weeks 200 g/d	Plasma and urine vitamin B1, B2 and B6,	The two yogurts: -↑plasma vitamin B1 _ plasma alteration of vitamin B2 metabolites (↓FAD, ↑FMN)	(85)
Coffee	B6, B12	NS	Interventional-RCT crossover	≥8 weeks	Netherland	Adults/18–60 years/male and female	26	Healthy	No-coffee period	1 L/day paper-filtered coffee, 4 weeks no-coffee period, 4 weeks	Plasma concentrations of vitamin B 6, B 12, and folate.	No significant changes in plasma B-6, B-12, or folate between coffee and no-coffee periods.	(87)

5-CH₃-H₄folate, 5-methyltetrahydrofolate; BMD, bone mineral density; cOC, carboxylated osteocalcin; CTX, C-telopeptide type I collagen; BAP, bone specific alkaline phosphatase; FAD, flavin adenine dinucleotide; FFQ, Food Frequency Questionnaire; FMN, flavin mononucleotide; Gla-OC, γ-carboxylated osteocalcin; Glu-OC, undercarboxylated osteocalcin; HbA1c, glycated hemoglobin; MK, Menaquinone (vitamin K2); NS, not specified; PGA, Pteroylmonoglutamic acid; PINP, procollagen type 1 N-terminal propeptide; RCT, Randomized Controlled Trial; RMR, Resting metabolic rate; RO, osteocalcin ratio; tOC, total serum osteocalcin. NS: not specified.

### 3.1 Fermented soybeans and cheese are valuable sources of vitamin K

#### 3.1.1 Food sources of vitamin K

Vitamin K exists as phylloquinone (K1) and menaquinones (K2, MK-4 to MK − 13), which differ in dietary sources, bioavailability, and function. Vitamin K1, primarily found in leafy greens and plant oils, accounts for the majority of dietary intake, but has limited bioavailability (34, 35). In contrast, vitamin K2, produced by bacterial fermentation, dairy products, and gut microbiota, exhibits longer half-life and enhanced extrahepatic distribution, making it particularly relevant for cardiovascular and bone health (35–38). Compared to vitamin K1, MK-7 and MK-9 exhibit prolonged half-lives, enhancing their bioavailability and ensuring sustained activation of vitamin K-dependent pathways (34, 39). Among menaquinones, MK-7, abundant in natto, is distinguished by its superior bioavailability and tissue retention, which is attributed to its longer unsaturated side chain (34). Swiss-type cheeses, such as Emmentaler and Jarlsberg, are rich in MK-9 and MK-9(4H), primarily due to the activity of *Propionibacterium freudenreichii* during cheese fermentation. Notably, Jarlsberg cheese was developed in Norway in the 1950s based on Emmental cheesemaking techniques, with researchers at the Agricultural University of Norway successfully adapting bacterial fermentation processes to create the cheese’s distinctive nutty flavor and characteristic holes via *P. freudenreichii* metabolism (40).

#### 3.1.2 Role of vitamin K and mechanism of action

The Adequate Intake (AI) for vitamin K, as established by the U.S. National Institutes of Health, is 120 μg/day for adult men and 90 μg/day for adult women (41). Vitamin K is essential for post-translational *γ*-carboxylation of glutamate residues in vitamin K-dependent proteins (VKDPs), a reaction catalyzed by γ-glutamyl carboxylase (GGCX) (Table 1) (34, 42, 43). This modification enables the activation of proteins involved in coagulation (factors II, VII, IX, and X), bone metabolism (osteocalcin), and vascular health (matrix Gla-protein, MGP) (44, 45). The vitamin K cycle, driven by vitamin K epoxide reductase (VKOR) and vitamin K reductase (VKR), ensures continuous recycling of vitamin K to maintain γ-carboxylation activity (42, 43). Beyond coagulation, vitamin K plays an integral role in bone metabolism, where it regulates osteocalcin carboxylation, a key factor in bone mineralization and skeletal integrity (44). The primary consequence of vitamin K deficiency is a bleeding tendency due to the relative inactivity of procoagulant proteins (2).

Vitamin K deficiency results in uncarboxylated osteocalcin, which impairs bone formation and increases fracture risk. Similarly, in the vascular system, MGP serves as a potent inhibitor of arterial calcification, preventing calcium deposition in soft tissues. Insufficient vitamin K leads to an accumulation of uncarboxylated MGP, which has been linked to vascular calcification and elevated cardiovascular disease risk (45). Studies indicate that K2 supplementation helps mitigate vascular calcification, reinforcing its importance in cardiovascular health (35). Emerging research suggests that vitamin K also plays roles in mitochondrial function, inflammation modulation, and neuroprotection, though further studies are needed to clarify its broader physiological impact (34, 35).

#### 3.1.3 Health effects of natto and cheese consumption

FF such as natto and Jarlsberg cheese are valuable dietary sources of vitamin K2. This review synthesizes findings from seven primary studies on natto (39, 46–51) and two on Jarlsberg cheese (52, 53), focusing on elderly individuals, premenopausal and postmenopausal women, and individuals with varied vitamin K status (Table 2).

While natto has been extensively studied, particularly in Japan, recent findings suggest Jarlsberg cheese – fermented with *P. freudenreichii—*may serve as an alternative vitamin K2 source in Western diets. However, comparative studies between these foods remain scarce, and the extent to which their different menaquinones [MK-7 vs. MK-9(4-H)] exert similar physiological effects remains unclear.

Natto is particularly rich in menaquinone-7 (MK-7), and serum MK-7 concentrations following natto intake have been shown to increase significantly and remain detectable for over 72 h, indicating prolonged bioactivity (49, 50). Tsukamoto et al. (50, 51) demonstrated that habitual natto consumption leads to a significant increase in serum MK-7 concentrations in a dose-dependent manner, with higher intake correlating with elevated *γ*-carboxylated osteocalcin (cOC) levels, particularly in men.

Sakamoto et al. (48) did not measure MK-7 levels but provided evidence that individuals with higher natto intake had increased plasma vitamin K concentrations and improved blood coagulation markers. Fujita et al. (39) found that elderly men with higher natto intake exhibited higher bone mineral density (BMD) at the total hip and femoral neck, with these effects likely mediated by vitamin K’s role in osteocalcin activation.

Several studies have established a dose-dependent relationship between natto intake and undercarboxylated osteocalcin (ucOC), a biomarker of vitamin K deficiency. Katsuyama et al. (47) reported that premenopausal women who consumed natto three times a week had significantly lower ucOC levels and higher bone-specific alkaline phosphatase (BAP) levels compared to those with lower natto intake. These findings suggest that natto promotes bone formation and may help maintain skeletal integrity before menopause.

Natto has also been linked to improved vascular health and regulation of coagulation. Schurgers and Vermeer (49) found that MK-7 from natto is associated with enhanced MGP activation. Tsukamoto et al. (50) showed that natto intake reduces plasma protein induced by vitamin K absence-II (PIVKA-II) levels, a marker of vitamin K deficiency, and lowers urinary calcium excretion, suggesting improved calcium retention.

For individuals taking the anticoagulant warfarin, natto consumption may pose potential negative interactions. Indeed, Homma et al. (46) demonstrated that consuming natto significantly increased plasma vitamin K concentrations, which could counteract warfarin’s anticoagulant effects. However, boiling natto markedly reduces its vitamin K bioavailability, potentially offering a method for patients on anticoagulation therapy to consume natto with a lower risk of drug interaction.

Jarlsberg cheese, a natural source of MK-9 (4H), has gained attention for its potential benefits to bone health and metabolism. A randomized controlled trial in premenopausal women demonstrated a significant increase in serum MK-9 levels and bone formation markers, particularly procollagen type 1 N-terminal propeptide (PINP), following regular consumption of Jarlsberg. When participants switched from Camembert (control) to Jarlsberg, their vitamin K2 levels and calcium absorption improved, suggesting a positive effect on bone metabolism (53). A follow-up study by the same group extended the intervention to 12 weeks, confirming sustained increases in PINP, total osteocalcin (tOC), and cOC, without elevating the bone resorption marker CTX. These findings support an anabolic effect on bone, possibly mediated by improved vitamin K2 status (52).

Jarlsberg cheese may also influence metabolism more widely. Indeed, in the same study, a reduction in glycated hemoglobin (HbA1c) suggests potential benefits for glucose regulation, though the underlying mechanisms are not yet clear (53). According to data by Walther et al. (54), Swiss cheeses, such as Emmentaler and Raclette, also contain notable amounts of long-chain menaquinones, with up to 549 μg/kg of MK-9(4H) in Emmentaler and 826 μg/kg of MK-7 in Raclette. However, to reach the dosages applied in clinical trials (180–200 μg/day), daily consumption would need to exceed 100 g, which may not be feasible for all populations due to high fat and salt content.

One possible factor is the presence of *P. freudenreichii*, a bacterial strain commonly found in cheese, which produces MK-9 and may contribute to vitamin K2 availability. However, unlike natto, the effect of MK-9 from cheese on blood clotting markers remains unclear due to a lack of direct intervention studies. More research is needed to determine whether MK-9 from dairy products offers the same cardiovascular and coagulation benefits as the menaquinones found in fermented soy.

The extended presence in circulation may explain why MK-7-rich natto and MK-9-rich cheese provide lasting physiological effects, particularly in populations at risk for osteoporosis and arterial calcification.

#### 3.1.4 Differences in responses and most relevant target groups

Variability in response to vitamin K2-rich foods has been observed across demographic groups. Men appear to exhibit a stronger correlation between MK-7 intake and *γ*-carboxylated osteocalcin activation, whereas older adults show greater improvements in bone density following natto consumption compared to younger individuals (39, 50). Individuals with low habitual vitamin K intake, particularly those consuming minimal FF, may experience more pronounced benefits from dietary interventions (34).

The strongest evidence for benefits exists among elderly individuals at risk for osteoporosis and vascular calcification, postmenopausal women experiencing accelerated bone loss, and individuals with vitamin K deficiency indicated by elevated PIVKA-II levels. Emerging research suggests potential applications for individuals with cardiovascular risk factors, as well as those with metabolic disorders such as diabetes, where reductions in HbA1c have been noted following Jarlsberg cheese consumption (53).

Despite strong evidence supporting the health benefits of natto and Jarlsberg cheese, several limitations remain. Most studies have focused on elderly individuals and postmenopausal women, limiting generalizability to younger populations (39, 47). Additionally, differences in dietary habits and gut microbiota may influence vitamin K metabolism and bioavailability (34).

While vitamin K2 is endogenously produced by gut bacteria, its systemic contribution to vitamin status is limited due to inefficient colonic absorption (34). Nonetheless, the gut microbiota plays an indirect role in vitamin K metabolism by modulating bile acid turnover and menaquinone production (35). Menaquinone profiles differ by microbial species: *P. freudenreichii* in Jarlsberg cheese synthesize MK-9 (4H), whereas *B. subtilis* in natto produces MK-7 (49, 53). These microbial contributions highlight the importance of FF as external sources of bioavailable vitamin K2.

Existing research has predominantly examined Japanese and European cohorts, leaving a gap in understanding how regional dietary patterns and microbiota-driven metabolism affect vitamin K bioavailability (35).

Further studies should investigate whether MK-7 from natto and MK-9 from cheese exert equivalent effects on bone and cardiovascular health, given their structural differences (49). Long-term intervention trials are needed to evaluate whether routine intake of vitamin K2-rich FF reduces fracture risk and cardiovascular disease incidence (44). Moreover, vitamin K2’s role in glucose metabolism and insulin sensitivity warrants further exploration (53).

Lastly, the potential of plant-based FF as alternative vitamin K2 sources should be investigated to meet the dietary needs of vegetarians and vegans, given their lower menaquinone intake, mainly present in animal-based products (34).

### 3.2 Folate (vitamin B9) produced during fermentation is able to contribute to an adequate folate status

#### 3.2.1 Food sources and forms of folates

Folate, also known as vitamin B9, is a generic term used for a family of compounds known as vitamers, differing by their oxidation state and the length of their glutamate residue. The chemical lability of all naturally-occurring folates results in a significant loss of biochemical activity during food storage and processing. In contrast, the synthetic form of this vitamin, folic acid, is a fully oxidized monoglutamate and the most chemically stable form (55). The bioavailability of natural folates compared to the synthetic form, folic acid, is variable and estimated to be between 10 and 98% (56). Folates are found in a variety of foods, including meat liver, green leafy vegetables, some other fruits and vegetables as well as legumes and cereals (as wholegrain products) (2). The majority of the population can meet the RDA with a daily intake of 400 μg of folate (2).

#### 3.2.2 Role and function of folates

Folate functions as a coenzyme or co-substrate in one-carbon transfers in the synthesis of nucleic acids (DNA and RNA) and metabolism of amino acids (16). Folate is also essential for the conversion of homocysteine to methionine and the synthesis of S-adenosyl-methionine, an important methyl donor for more than 100 methyltransferases for a wide range of substrates such as DNA, hormones, proteins, neurotransmitters and membrane phospholipids, all of which being regulators of important physiological processes (55). Folate deficiency impairs DNA replication and cell division, that can lead to megaloblastic anemia, or neural tube defects (Table 1) (57).

#### 3.2.3 FF sources of folates in human studies

Despite the high number of articles dealing with the increase in folate content of food using folate-producing microorganisms, only three human studies were identified in the literature, two on camembert cheese, and one on tea (Table 2).

The definition of dietary folate equivalents is generally based on the assumption of 50% bioavailability (58). The aim of Mönch et al. (59) work was to assess the relative bioavailability of folates in several folate-rich foods, including low-fat camembert cheese. They conducted a short-term human study measuring plasma folate levels after ingestion of the foods using an isotopic dilution assay. This demonstrated that folate from the tested foods including camembert was absorbed with matrix-dependent bioavailability and with high interindividual variations, thus showing that total folate concentration in food does not always correlate with its bioavailability. The authors also measured the folate concentration of the most abundant vitamers in low-fat camembert from different brands, to investigate the potential variation in folate bioavailability within the same food. Folate concentration in camembert ranged from 224 to 647 nmol/100 g (59, 60), which is 5 to 15 times higher than in milk (61) if calculated for dry weight. Moreover, a significant variation in folate bioavailability has been reported depending on the cheese brand. Notably, Mönch et al. (59) observed that low-fat camembert in their study exhibited substantially higher folate bioavailability (65–71%) compared to the 8.8% reported in a previous study on a different brand (60). Despite containing a lower total folate content (224 nmol/100 g) compared to the previously studied camembert (647 nmol/100 g), the cheese analyzed in this study presented a distinct folate profile. Specifically, 5-formyltetrahydrofolate was the predominant vitamer, as opposed to tetrahydrofolate in the higher-folate cheese. An intraindividual comparison of participants who participated in both studies further confirmed the striking difference in bioavailability between the two cheese brands. These findings highlight that fermentation processes, cheese composition, and folate vitamer distribution significantly influence folate bioavailability, reinforcing that a food product (e.g., camembert cheese) cannot be considered a homogeneous dietary standard with predictable folate bioavailability (59).

Several hypotheses have been proposed regarding the differences in folate bioavailability, including differences in the kinetics and bioavailability of folate vitamers, particularly polyglutamate forms; the presence of deconjugase inhibitors and entrapment of folates within the food matrix (60).

Tea can also be a source of folates, reaching up to 7 μg/100 mL of brewed tea (62). A positive correlation has been observed between tea consumption and plasma folate concentration in China, with specificity varying by ethnic group and tea type. Han women, drinking unfermented tea weekly, and Uighur women, drinking fully fermented tea weekly, exhibited this positive correlation; however, the role of fermentation was unclear and deserves further research (63).

Although many articles are dedicated to the study of fermentation as a way to improve folate content of FF, the lack of human studies together with the large differences in the bioavailability of vitamin B9 due to a food matrix effect or the vitamers present in the FF, leads to under- or overestimated the impact of FF-enriched food on the folate status of populations. Further human studies would help to better understand how these FF could be used to improve vitamin B9 status, especially for vulnerable populations at risk of deficiency, such as young children and women of childbearing age.

### 3.3 FF as source of the other vitamin B (B1, B2, B3, B5, B6, B12)

In addition to vitamin B9, other vitamin Bs can be produced by different microorganisms during the fermentation of various foods. However, this capability is strain-specific, as microorganisms can either synthesize or consume them. Five studies were dedicated to the effect of FF consumption on vitamin B status (Table 2).

#### 3.3.1 Food source and roles of the other vitamin B

Thiamine (vitamin B1) is found in whole grains, legumes, nuts, and meat. The RDA is 1.6 mg for adult males. A deficiency can result in conditions such as beriberi or Wernicke-Korsakoff syndrome, which affect the nervous system (64). Vitamin B1 is an essential cofactor involved in carbohydrate metabolism, the respiratory chain, amino acid catabolism, and nucleic acid biosynthesis. In addition, thiamine plays a neuro-modulatory role by supporting acetylcholine neurotransmission and helps maintain neuronal and cellular membrane integrity (65). Recently, LAB strains have been screened for their ability to produce this vitamin (Table 1) (66).

Food sources of riboflavin (vitamin B2) include liver, eggs, dark green vegetables, legumes, whole and enriched grain products, milk, and fermented products (67). In recent years, different lactic acid bacteria selected for their ability to overproduce this vitamin have been proposed for the *in situ* biofortification of various FF (68–71). The RDA is 1.4 mg for adult males. Ariboflavinosis can cause symptoms such as cracked lips, a sore throat, and skin disorders. Vitamin B2 is the precursor of the coenzymes flavin mononucleotide (FMN) and flavin adenine dinucleotide (FAD) that are involved in critical metabolic pathways such as the cycle of Krebs, redox reactions, as well as in the biosynthesis of other vitamins (Table 1).

Dietary niacin (vitamin B3) is primarily found in meat, poultry, fish, eggs, dairy products, nuts, seeds, whole grains, legumes, and certain vegetables. The RDA is 16 mg/day for adults. A deficiency of niacin leads to pellagra, a condition marked by dermatitis, diarrhea, and dementia (72). Vitamin B3 act as precursor of nicotinamide adenine dinucleotide (NAD) and NAD phosphate (NADP), both of which play crucial roles in energy production and redox reactions. Recent studies highlighted its anti-inflammatory and antioxidant properties. Additionally, this vitamin helps regulate blood cholesterol levels, particularly by boosting high-density lipoprotein (HDL) cholesterol (Table 1) (73, 74).

Pantothenic acid (vitamin B5) is primarily found in meat, eggs, dairy, whole grains, legumes, avocados, some vegetables and mushrooms. The RDA is considered to be between 5 and 7 mg for adults. Vitamin B5 plays a crucial role in metabolizing carbohydrates, proteins, fatty acids. In recent years, D-pantothenic acid has been investigated for its potential in treating neurodegenerative diseases (Table 1) (75).

The richest sources of pyridoxine, pyridoxal and pyridoxamine (vitamin B6) include fish, beef liver and other organ meats, potatoes and other starchy vegetables, and fruit. The RDA is about 2.0 mg/day for adults. Vitamin B6 deficiency is linked to several pathological conditions, including end-stage renal disease, chronic renal insufficiency, and other kidney-related disorders. Moreover, an insufficient intake of this vitamin can lead to malabsorption syndromes, such as celiac disease and inflammatory bowel diseases, including Crohn’s disease and ulcerative colitis (76). Vitamin B6 plays a role in various biological processes, including amino acid, sugar, and lipid metabolism (Table 1) (77).

Cobalamin (vitamin B12) is the only vitamin exclusively from microbial origin, since synthesized by some bacteria and archaea. Cobalamin is present in foods of animal origin, due to the production by microorganisms present in the digestive tract, which is absorbed and incorporated into animal tissues. Thus, animal-sourced products such as meat, fish, eggs and dairy products are main dietary source of cobalamin (WHO, 2004). RDA between 3.8 and 20.7 μg is required to maintain adequate levels and prevent deficiency in healthy adults and older individuals (6). Among bacteria of food interest, *Propionibacterium* spp. is well known for producing cobalamin to varying extents (78). This capability has been reported also in some strains of lactic acid bacteria (LAB) (79). However, this property remains controversial, as LAB often synthesize corrinoid compounds (pseudo-cobalamin) that are biologically unavailable to humans (80). A deficiency in this vital nutrient can lead to both hematological and neurological disorders, including the serious condition known as pernicious anemia (81). Vitamin B12 is essential in cellular metabolism, especially in DNA synthesis, methylation and mitochondrial metabolism (Table 1).

#### 3.3.2 FF consumption contributes to the vitamin B status of different population

Five studies were identified as addressing the effect of consuming some FF on the nutritional status of these metabolites, and all of them tackled different vitamin B deficiencies simultaneously. Plasma or urine metabolomes analysis have been recently used to detect biomarkers from FF consumption (Table 2).

In a prospective cohort study of Dutch men and women (20 to 70 years), non-targeted metabolomics analyses were performed on plasma and urine samples (*n* = 485 and *n* = 492, respectively) of a subcohort of participants (*n* = 531) (82). Urinary niacin (vitamin B3) has been reported as the unique vitamin B candidate biomarker of habitual coffee intake and the other vitamin B (B2 and B5) also present in coffee were not identified (82). Whether the fermentation of coffee is responsible for its vitamin B content remains to be clarified. A recent cohort study conducted within the Framingham Heart Study Offspring (3,712 participants, average age ~55 years) and third generation cohorts (4,078 participants, average age ~40 years) utilized both targeted and untargeted biomarkers to analyze plasma metabolomes associated with quantity and type of dairy intake (83). A link between dairy consumption and the circulating levels of pantothenate (vitamin B5) and niacin (vitamin B3) was established. Specifically, pantothenate concentrations demonstrated a statistically significant association with overall dairy intake in the third-generation cohort, aligning with observations in the offspring cohort. However, correlation with milk only was not significant, suggesting that fermentation processes might contribute to increased vitamin levels. Notably, plasma pantothenate displayed a statistically significant and directionally consistent association with cream and butter in the offspring cohort (83).

Two interventional studies investigated the effect of either the consumption of traditional yogurt fermented with *Streptococcus thermophilus* and *Lactobacillus acidophilus* or probiotic yoghurt fermented with *Lacticaseibacillus casei* GG in addition, on thiamine (vitamin B1), riboflavin (vitamin B2), and pyridoxine (vitamin B6) status in healthy adults (84, 85). The first study aimed to determine whether viable probiotics in yoghurt could influence the status of B1, B2, and B6 vitamins in healthy adult humans; either by using or producing these vitamins (84). Thus, 12 volunteers (six healthy males and 6 healthy females, aged 25–36 years) consumed 500 g of yoghurt daily during 4 weeks. They received yoghurts containing thermally inactivated cultures during the first 2-week period and yoghurts not submitted to heat treatment during the second 2-week period. Thiamin levels in plasma significantly declined after the first 2 weeks and continued to decrease during the second period,. Thiamine urinary excretion and B2 metabolites were also affected. Indeed, FAD increased significantly after consuming heat-treated yogurt but dropped after switching to untreated yogurt. Conversely, FMN levels decreased initially but rose later, though not significantly. Free riboflavin in plasma and urine showed a slight, non-significant increase. Pyridoxal-5-phosphate (vitamin B6) levels increased with heat-treated yogurt but decreased afterwards, with no significant differences observed. Therefore, these results suggest that the presence of *Lacticaseibacillus casei* GG as a probiotic in yoghurt would reduce the bioavailability of vitamins B1, B2 and B6, despite the fact that it was known to produce thiamine, riboflavin and folate in culture medium (84, 86). Investigation of the potential vitamin B-producing capabilities of the bacteria used in yogurt preparation was not shown. In a similar approach by the same research team with lower amounts of yogurt consumption and another probiotic strain (*Lacticaseibacillus casei* subsp. *casei*) were used. This intervention study was conducted in young healthy Austrian women (*n* = 33) with the aim to investigate whether probiotic and/or traditional yoghurt bacteria were able to influence the status of B1, B2 and B6 vitamins (85). The results indicated that daily consumption of 200 g of both probiotic and conventional yoghurt for 2 weeks resulted in the increase levels of plasma thiamine and free riboflavin, while the urinary excretion of these vitamins remained unaffected. Interestingly, the plasma concentration of FAD decreased significantly after consuming 100 g of yoghurt/day, while, conversely, the plasma levels of FMN and free riboflavin increased significantly, suggesting a different physiological role of these flavins. The average status of vitamin B6 was unaffected by yoghurt intake in both plasma and urine. Overall, the authors concluded that the reported variations in plasma vitamins levels were more likely consistent with yoghurt consumption as a fermented dairy product rather than by the specific ingestion of probiotic bacteria (85). These two studies on a limited number of volunteers, differing in sex, the amount of FF and the bacterial strains used, gave different results, underlying the need to evaluate the effect of FF consumption enriched in vitamins by fermentation in human on a larger scale.

One study investigated the link between coffee consumption and levels of certain B-vitamins in the blood. The impact of daily consumption of 1 L of paper-filtered coffee on plasma total homocysteine concentrations was assessed in healthy subjects through a randomized, controlled, and crossover intervention (87). Briefly, 26 healthy participants (male and female, aged 18–60 years) were involved in a 4-week study (coffee consumption and no-coffee period) with a crossover design. This study also monitored plasma concentrations of vitamin B6, vitamin B12, and folate, as their deficiencies might be related to elevated homocysteine concentrations. However, no significant differences were found between the coffee and no-coffee periods (87). Nevertheless, no information on the vitamin B6 and B12 content of coffee were provided.

Surprisingly, no studies were found on the effect of FF enriched in vitamin B12 by fermentation in humans. Vitamin B12 is absent from non-animal-based products, and many people in the world consume no or little of this food category due to affordability or personal choices; therefore, this topic deserves specific attention. In addition, different microorganisms encountered in FF may produce pseudo-cobalamin, which is inactive in humans. Intervention studies using FF fortified in vitamin B12 are obviously required.

### 3.4 Vitamin A and D, the left aside of vitamin production through fermentation

#### 3.4.1 Vitamin A is produced by a few microorganisms not present in FF

Vitamin A is naturally present only in animal-derived foods, while many fruits and vegetables can provide carotenoids that can be converted into vitamin A at intestinal level (15). The main dietary sources of vitamin A include liver, dairy products, and fish, while provitamin A carotenoids are abundant in colorful fruits and vegetables, such as carrots, sweet potatoes, and spinach. The RDA of vitamin A is 900 and 700 μg/day for adult men and women, respectively. Vitamin A is an essential nutrient best known for supporting vision and regulating cell function, proliferation, and tissue homeostasis. A deficiency can result in poor nutritional status, increased mortality risk, weakened overall health, reduced tissue growth, slower metabolic processes, and heightened susceptibility to infections (15).

Different microorganisms belonging to *Erwinia* or *Agrobacterium* genera among others, can also synthesize vitamin A or provitamin A, but only a few of them are present in FF (20). Microorganisms may also modulate carotenoids bioavailability (26). Only one study dealt with the effect of ingestion of fermented orange juice or its non-fermented counterpart on carotenoid profile on blood carotenoids. Bioavailability of carotenoids was higher when orange juice was fermented. However, carotenoids were most probably coming from the orange juice itself, and not from the action of microorganisms since carotenoid profiles were similar in the two juices. The yeast species used for fermentation, *Pichia kluyveri* var. *kluyveriis* is not known for its carotenoid producing capability (88).

#### 3.4.2 Vitamin D can be produced by *Saccharomyces cerevisiae*, but is not used to increase vitamin D content in FF

Vitamin D, also referred to as calciferol, is a fat-soluble vitamin that can either be synthesized endogenously in the skin following exposure to UV-B irradiation or can be provided from food sources and dietary supplements. The version made in the skin is referred to as cholecalciferol (vitamin D3) whereas the dietary form can be vitamin D3 or a plant-origin compound known as ergocalciferol (vitamin D2). Vitamin D obtained from sunlight, food, and supplements is initially biologically inactive and must undergo two hydroxylation processes in the body to be converted into its active form, 1,25-dihydroxyvitamin D [1,25-(OH)2D], also known as calcitriol. The RDA of vitamin D is 400 and 800 μg/day for adult male and elderly, respectively (2, 16).

Vitamin D, through its hormonal form calcitriol is a central regulator of calcium and phosphate homeostasis, which is necessary for normal bone mineralization, muscle function, nerve conduction, and overall cellular activity in the body (2, 16). Vitamin D also plays various other roles in the body, such as reducing inflammation and modulating processes like cell growth, neuromuscular function, immune responses, and glucose metabolism (16). Additionally, vitamin D has immunomodulatory effects that may alter responses to infections *in vivo*. These properties, which include promoting cell differentiation and modulating immune function, are why vitamin D derivatives are effectively used in the treatment of various skin conditions. Adequate vitamin D levels prevent rickets in children and osteomalacia in adults, while also helping protect older individuals from osteoporosis (16).

Literature reports the capability of yeasts, particularly *Saccharomyces cerevisiae*, to synthesize ergosterol, one of the precursors of vitamin D, for industrial production, but literature on the use of yeast to increase vitamin D content of FF and their possible contribution to vitamin D intakes is not available (19). The few studies dealing with the ingestion of vitamin D from FF are from dairy products, where the vitamin D is added through fortification (89, 90). Whether fermentation can be used to increase the vitamin D content in food and improve dietary intake of vitamin D remains to be explored.

### 3.5 Detailed FF characterization is key for essentiality of nutrients

FF characterization is necessary to be able to ascertain the scientific evidence of health claims based on FF enriched in vitamin by fermentation (30). Different factors may influence the physiological effect of vitmains, including batch-to-batch variability, their concentration in FF as well as difference in chemical structure (vitamers, precursors) and bioavailability. The study on camembert cheese is one of the rare examples where the major vitamin B9 vitamers were quantified in the FF and in the plasma after ingestion of the FF (59, 60). The different bioavailability patterns and the important individual observed variation underscore the need to implement similar studies in different food matrixes, with various fermentation types, in order to verify the real contribution of the FF enriched in vitamin by fermentation at a large scale.

In addition, the formulation of the FF should be specified and this is particularly relevant since the food matrix may influence the absorption of the vitamin. Indeed, in the same example, folate from spinach was better absorbed than folate from cheese and different hypotheses were raised to explain this result (see corresponding paragraph). This example highlights the need to evaluate many FF enriched in vitamins through fermentation to ascertain the efficiency of the strategy in different contexts.

The variability between individual foods, which may influence the vitamin content of the FF should also be characterized. Of the 18 studies discussed in the present paper, only two, on cheese and vitamin B9, investigated the possible differences between two brands of the same cheese and highlighted significant differences. This variability would be even more important to assess in the case of FF with non-controlled fermentation (spontaneous, backslopping, initial high endogenous microbiota), since the microorganisms involved differ from one fermentation to another. As microorganisms may produce or consume vitamin B9, the overall production/consumption balance should be assessed. Indeed, many traditional foods prepared using spontaneous or backslopping fermentation at room temperature exhibit almost at total disappearance of folate initially present in the raw material or a huge increase in the vitamin B9 content, able theoretically to cover a large part of the RNI (30%) (91, 92).

Where controlled fermentation with known microorganisms is used, sufficient information must be provided, in addition to the identification of the microbial species, to enable the control authorities to carry out their own monitoring (30). On the 18 studies presented here, only five specified the species used for fermentation.

More generally, while the FF category is almost always indicated (dairy products, fermented legumes, etc.), the precise description of the food is almost never sufficient. For example, ingredients (and even less their composition) are not necessarily mentioned, the process is not always described. Although, the food safety of the FF presented in this review is never mentioned, we can hypothesize that in interventional studies, the ethical approval included this aspect and that it is difficult to assess it from observational studies.

### 3.6 Role of microbiota to improve vitamin status

Microorganisms in the gut can influence the host’s vitaminic status by synthesizing vitamin or regulating their absorption. Notably, bioavailability appears to be enhanced by the production of short-chain fatty acids, which are the end products of microbial polysaccharide fermentation (93). In turn, dietary vitamin intake can modulate the composition and functionality of the microbiome, which in its turn influences bioavailability of vitamins. Microorganisms in FF could also synthesize vitamins while transiting through the digestive tract. If the role of gut microbiota is well documented for vitamin K (see corresponding section), it is not the case for all B vitamin.

#### 3.6.1 Synthesis of vitamin by microorganisms in the gut

While most vitamins are obtained from food, some can be synthesized by gut bacteria and absorbed in the colon (94). The role of intestinal bacteria in vitamin K production has been recognized for decades, and their contribution to other vitamins synthesis has also been documented (26). Bacterial synthesis may provide 10–50% of the body’s vitamin K requirements, though the exact proportion remains uncertain (11). Most menaquinones (MK-n) are produced in the colon without bile salts, raising questions about their absorption, as bile salts are typically required for vitamin K uptake in the small intestine. If absorbed, MK-n from colonic bacteria likely follows a bile-salt-independent route, whereas those produced in the ileum—a smaller fraction—may be absorbed via a bile-dependent pathway (95). However, the bioavailability of MK-n from gut bacteria is limited, as they remain within bacterial membranes rather than being freely available (96).

Data on the role of intestinal bacteria from the gut on vitamin B productions is less documented. Metagenomic analyses have revealed that some bacteria, such as *Bacteroidetes* and *Actinobacteria*, possess the genetic potential to synthesize vitamin B1, whereas most *Firmicutes* do not (97). *Lactobacillaceae* and *Bifidobacteriaceae* produce riboflavin, which may be utilized by colonic epithelial cells, though its exact contribution *in vivo* remains unclear (98, 99). While riboflavin biosynthesis has been well studied in *B. subtilis* and *Escherichia coli*, a systematic genome analysis of 256 human gut strains found that 40–65% could theoretically produce one or more B vitamins (B1, B2, B3, B5, B6, B7, B9, and B12) (100). Complete folate biosynthesis pathways were detected in nearly all *Bacteroidetes, Fusobacteriota* (syn. *Fusobacteria*), and *Proteobacteria* genomes, whereas *Actinobacteria* and *Firmicutes* often lacked the para-aminobenzoic acid biosynthesis pathway (26).

The *de novo* synthesis of cobalamin (vitamin B12) is an intricate process that involves over 30 enzymatic steps and more than 60 gene families (101). This capability is exclusive to certain bacteria and archaea. Transporters of vitamin B12 are present only in the upper parts of the digestive tract. Therefore, the eventual synthesis by bacteria from the colon should not contribute significantly to the vitamin B12 status of the host (102). LAB are present in higher relative abundance in the small intestine, but to our knowledge, no studies have investigated their eventual role in contributing to cobalamin absorption.

#### 3.6.2 Does vitamin consumption enhance gut microbiota–mediated vitamin biosynthesis/ absorption?

Within the human gut microbiota, increased folate intake has been positively correlated with a higher abundance of *Faecalibacterium, Akkermansia,* and *Roseburia* genera, as observed in mucosal and colonic biopsy samples (103). Furthermore, carotenoid intake and status have been linked to increased microbial diversity and a rise in beneficial bacteria. The most relevant studies on the impact of carotenoids on gut microbiota have been reported by Bernabeu et al. (104). There is a strong evidence that carotenoids exert a prebiotic effect, promoting the growth of specific bacterial species such as *Lachnospiraceae, Bifidobacterium,* and *Prevotellaceae*. Park et al. (105) demonstrated that vitamin B1 significantly influences the survival and competition of bacteria within the gut microbiota. They also showed that vitamin B1 plays a role in butyrate production and contributes to the regulation of acetate levels in the intestinal environment. Additionally, microbial riboflavin production is influenced by a fiber-rich diet (106). Bacteria require folate for growth: some are prototrophic and can synthesize it from environmental precursors, while others, known as auxotrophic bacteria, must acquire it directly from their surroundings. These findings suggest that the gut microbiota is a key source of folates and that changes in its composition—driven by factors such as fiber consumption—may affect folate requirements (107, 108).

Considering that microorganisms can produce and use vitamin, an interplay between microorganism for vitamin occurs. This has been demonstrated, for example, in a synthetic co-culture experiment using bacteria from a human fecal sample, where differences in cross-feeding were observed (109). The bacteria in the gut are also able to capture and differentiate vitamin B12 analogs, competing in the gut (110).

On the whole, the contribution of gut microbes to vitamin status and the effect of FF consumption on vitamin synthesis in the gut remains to be studied more specifically.

## 4 Perspectives, gaps and conclusion

This systematic narrative review highlights the potential of FF as a natural way for enhancing vitamin intake via microbial production. However, significant gaps remain in understanding the effectiveness and reliability of this approach (Table 3). The bioavailability of vitamins from FF varies depending on the microbial strains, fermentation conditions, vitamers produced, and the food matrix involved. There is a notable lack of human studies on several essential vitamins, including B12, which is particularly important for population consuming no or low animal-based products. These sources of variation are often overlooked when vitamin intakes are estimated through food consumption surveys, which can lead to a significant misestimation of vitamin intakes. An example from Ethiopia showed that the sampled population had totally inadequate dietary intakes of vitamin B12, but only 25% of the population had low serum cobalamin levels (111). One hypothesis is that high consumption of FF and the role of fermentation, which has been estimated but not experimentally validated (112).

**Table 3:** Overview of the FF, microbiota and summary of the findings.

FF	Vitamin	Population	Vitamin producing microorganism	Vitamin concentration in food	Plasma biomarkers	Vitamin related health effect	Role of gut microbiota
Natto	K2 (MK-7)	Healthy	*Bacillus subtilis* var. natto (specified in 4/7 studies)	Specified in 7/7 studies	Measured	Measured	Not studied
Cheese	K2 [MK-9, MK-9 (4H)]	Healthy	*Propionibacterium freudenreichii* (specified in 1/2 studies)	Specified in 2/2 studies	Measured	Measured	Not studied
	B9	Healthy	Not studied	Specified in 2/2 studies	Measured	Not studied	Not studied
Tea	B9	Healthy	Not studied	Not studied	Measured	Not studied	Not studied
Yogurt/fermented milk	B1, B2, B6	Healthy	Specified in 2/2 studies	Not studied	Measured	Not studied	Not studied

The wide variety of FF around the world, the microorganisms responsible for their fermentation and their potential contribution to vitamin intakes has also been underexplored. Many microorganisms very well known for their vitamin synthesis capabilities, such as *Saccharomyces cerevisiae*, which can produce vitamin B9 in large amount, have not been studied in humans. All the data presented in this review are from Europe, Asia, and the USA, excluding other parts of the world that also consume various FF, with potentially different vitamin profiles (9). Populations at risk of deficiencies include young children, women of childbearing age and the elderly, and future studies should include these different categories. This wide variety of FF may a serve as a source of inspiration for one country to learn from another, utilizing the microbial diversity encountered in these traditional FF. These FF can be used as single strains but also combined to explore cumulative vitamin production by using microorganisms with different vitamin potentials together. Indeed, examples of the use of combination of strains with synthesis vitamin capabilities are rare, and not always efficient (113).

Numerous studies lack comprehensive information regarding the composition, safety, and microbiological content of the examined FF. To endorse FF-based vitamin fortification as a public health strategy, future research should concentrate on well-designed human intervention studies, thorough evaluations of vitamin variants and food matrices, and uniform documentation of fermentation methodologies and microbial strains. Moreover, the impact of the gut microbiota on vitamin status, encompassing vitamin synthesis, absorption, and microbial cross-feeding, remains inadequately defined. Addressing these gaps will enable better assessment of the nutritional impact of FF and guide the development of evidence-based dietary recommendations.

## Data Availability

The original contributions presented in the study are included in the article/supplementary material, further inquiries can be directed to the corresponding authors.
